# Customizing Properties of β-Chitin in Squid Pen (Gladius) by Chemical Treatments

**DOI:** 10.3390/md12125979

**Published:** 2014-12-15

**Authors:** Alessandro Ianiro, Matteo Di Giosia, Simona Fermani, Chiara Samorì, Marianna Barbalinardo, Francesco Valle, Graziella Pellegrini, Fabio Biscarini, Francesco Zerbetto, Matteo Calvaresi, Giuseppe Falini

**Affiliations:** 1 Department of Chemistry “Giacomo Ciamician”, Alma Mater Studiorum, University of Bologna, via Selmi 2, 40126 Bologna, Italy; E-Mails: alessandro.ianiro@gmail.com (A.I.); matteo.digiosia2@unibo.it (M.D.); simona.fermani@unibo.it (S.F.); chiara.samori3@unibo.it (C.S.); rancesco.zerbetto (F.Z.); 2 National Research Council (CNR), Institute for Nanostructured Materials (ISMN), Via P. Gobetti 101, 40129 Bologna, Italy; E-Mails: m.barbalinardo@bo.ismn.cnr.it (M.B.); f.valle@bo.ismn.cnr.it (F.V.); 3 Centre for Regenerative Medicine “Stefano Ferrari”, University of Modena and Reggio Emilia, Via G. Gottardi, 100, 41125 Modena, Italy; E-Mail: graziella.pellegrini@unimore.it; 4 Department of Life Sciences, University of Modena and Reggio Emilia, Via Campi 183, 41125 Modena, Italy; E-Mail: fabio.biscarini@unimore.it

**Keywords:** β-chitin, hierarchical structure, squid pen, deacetylation, mechanical properties, wettability, porosity

## Abstract

The squid pen (gladius) from the *Loligo vulgaris* was used for preparation of β-chitin materials characterized by different chemical, micro- and nano-structural properties that preserved, almost completely the macrostructural and the mechanical ones. The β-chitin materials obtained by alkaline treatment showed porosity, wettability and swelling that are a function of the duration of the treatment. Microscopic, spectroscopic and synchrotron X-ray diffraction techniques showed that the chemical environment of the *N*-acetyl groups of the β-chitin chains changes after the thermal alkaline treatment. As a consequence, the crystalline packing of the β-chitin is modified, due to the intercalation of water molecules between β-chitin sheets. Potential applications of these β-chitin materials range from the nanotechnology to the regenerative medicine. The use of gladii, which are waste products of the fishing industry, has also important environmental implications.

## 1. Introduction

Natural bio-products and processes are becoming more and more important for the preparation of new materials [1]. In biologically inspired synthetic procedures *top down* and *bottom up* approaches are possible [2]. The synthesis of a biological material following the *bottom up* approach represents a difficult target to achieve; this is due to the complexity of biological materials that entail many processes, hierarchically controlled and orchestrated in space and time at the cellular level. The *top down* approach, where breaking down of a complex material allows the use of the properties of its sub-units, implies the possible loss of some peculiar properties that are obtained by the controlled assembly. A different approach can be the molecular, mesoscale, chemical modification of natural materials (*up*) under the constraint of preserving as much as possible their intrinsic structure and/or hierarchical organization (*top*). In this *top up* approach, the characterizing macro-properties of the natural materials are preserved, while other features can be modified at the micro- and nano-scale, according to specific needs.

Examples of the *top up* approach include:

(a) spines of the echinoderms that were hydrothermally treated, converting calcium carbonate in calcium phosphate, preserving the spine microporous structure, and making them useful as bone graft substitutes [3],
(b) nanometric silk-fibroin nets, fabricated by electrospinning from regenerated *Bombyx mori* silk-fibroin that were made water-insoluble by the reaction with genipin that induced conformational changes favoring the presence of a beta-sheet together with beta-turn intermediates [4],
(c) natural biosorbent obtained from *Pyracantha coccinea* that was modified with an anionic surfactant to facilitate its dye removal ability,
(d) a modified biosorbent that was successfully employed for the decoloration of methyl violet contaminated solutions [5],
(e) periodic superconducting, porous self-supporting monoliths that were synthesized using cuttlebone as a morphological template; this produced a lightweight, structurally stable superconductor with a greatly improved critical current density [6].

Among the natural materials modifiable by the *top up* approach, the chitinous ones appear very promising [7,8]. Chitin is widely used in nature to build complex hierarchical structures with optimized functional properties. It is the main component in crab and shrimp shells, in the outer skins or cuticles of other arthropods, and is present in the molluscan shell, coexisting with proteins and minerals [9]. From the chemical and structural points of view, chitin consists of β-(1-4)-linked *N*-acetyl anhydroglucosamine polymeric chains and shows mainly α- and β-polymorphs. In α-chitin, all molecular chains are arranged in an antiparallel mode with strong intermolecular hydrogen bonding. β-chitin has a parallel chain packing with intermolecular forces weaker than those between the chains of α-chitin. This feature makes β-chitin more susceptible to enzymatic degradations or chemical reactions [10,11,12,13]. Most natural chitins have α-type structure, while β-type chitin is less diffuse [9].

Among chitinous materials the native squid pen (gladius), a waste product of fishing industries [14], has been widely used after dissolution and deacetylation as a source of chitosan for the preparation of many different materials [15]. The gladius, a feather-shaped internal structure that supports the squid mantle, serves as a site for muscle attachment. It presents features that can be modified by applying a *top up* approach. The gladius is constituted mainly of proteoglycans and β-chitin [16]. In the hierarchical structure of the gladius the β-chitin crystallites [17] are wrapped in a protein layer and form nano-fibrils. These are the building blocks of 0.2 µm sized micro-fibers. The fibers aggregate into 2 µm, 10 µm, 100 µm and 500 µm thick fibers, which eventually form the gladius [18]. The crystalline structure of β-chitin has been reported [19]: its unit cell is monoclinic, spacegroup P2_1,_ with dimensions of a = 4.85 Å, b = 9.26 Å, c = 10.38 Å (fiber axis), and γ = 97.5°. The structural study showed that anhydrous β-chitin adopts a molecular sheet in the *a c* plane that is formed by hydrophobic forces of glucopyranoside rings and by intermolecular hydrogen bonds C=O···H–N and C=O···H–O6. These sheets are stacked because of the presence of hydrophobic forces, and thus no hydrogen bond exists between the sheets along the (010) direction [19].

In this work, the structural organization of β-chitin in the gladius is studied at different length scales after deproteinization and a partial deacetylation. The latter process is carried out preserving as much as possible the overall ultrastructural organization of β-chitin chains. The goal is to prepare bio-materials with additional tunable chemical properties over the ultrastructural and mechanical ones of the β-chitin of the gladius. Possible applications of chitin in different fields include cosmetics, agricultural products, waste water treatment, functional food, drug delivery and regenerative medicine. While the natural functions of chitin are mostly structural, their applications were focused on the exploitation of its chemical properties rather than its ability to form structures.

The *top up* approach can overcome current limitations for the applications of chitin materials, but at the same time shows some restrictions. The use of chitinous materials for structural applications has been limited by the inability to reproduce the complex hierarchical design of naturally occurring chitin composites. Application of the *top up* approach preserves the hierarchical structure of chitin. This protocol developed here offers a solubilization-free procedure. In addition, the use of the gladius as a source of a multiscale structured of β-chitin does not require a demineralization step since the low content of inorganic components and the removal of the protein is simpler than for other chitinous sources.

The *top up* approach on the gladii is relevant also for environmental issues. The gladii are natural waste products from fishing industries and their disposal has significant costs [14]. According to the Food and Agriculture Organization, the squid catch for 2002 was above 2 Mtonnes [20]. Despite a number of advantages, some crucial aspects can still affect the results of the *top up* approach. Among them, the difficulty to receive reproducible batches of product from various sources of raw materials, the collection of raw materials of satisfactory quality, and the absence of standardization of product quality and product assay methods for chitin and its derivatives if the scope is to provide medical applications.

## 2. Results and Discussion

**Figure 1:**
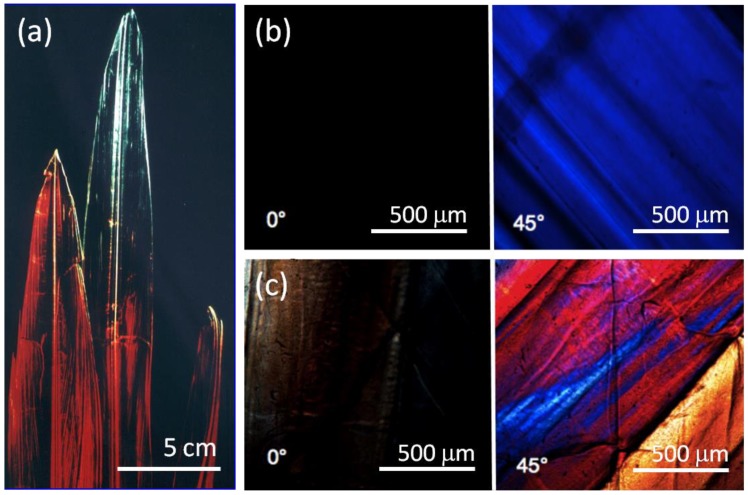
(**a**) Camera image of three *Loligo vulgaris* squid pens (gladii) illuminated using polarized light. The gladii show dichroic effect; (**b**) Crossed polarizer optical micrograph of a blade region of untreated gladius sample under crossed polars (0°) and after rotation of one polar of 45°; (**c**) Crossed polarizer optical micrograph under crossed polars (0°) of a blade region of a gladius sample treated for 2 h in a boiling 1.0 M NaOH solution and after rotation of one polar of 45°.

**Figure 2:**
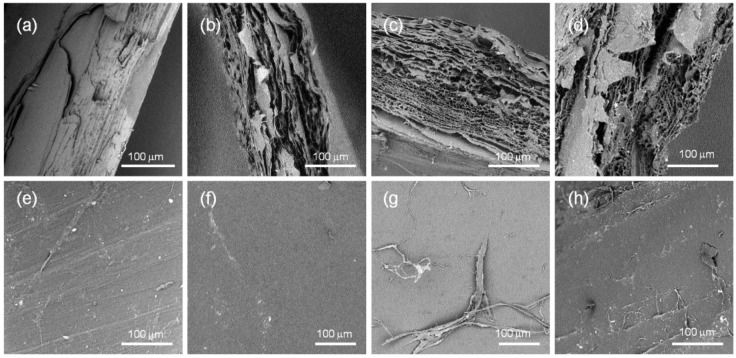
Scanning electron microscopy images of *Loligo vulgaris* gladius samples. (**a**,**e**) Untreated sample; (**b**,**f**) Sample treated for 2 h in a boiling 1.0 M NaOH solution; (**c**,**g**) Sample treated for 6 h in a boiling 1.0 M NaOH solution; (**d**,**h**) Sample treated for 24 h in a boiling 1.0 M NaOH solution; (**a**–**d**) Cross section view; (**e**–**h**) Surface view of the blade region.

The central rib and lateral blades of the gladius from *Loligo vulgaris* shows a high degree of birefringence and colors, when illuminated with polarized light (Figure 1a), because of the presence of a high degree of fibers alignment. The material does not transmit light under optical microscope when the polars are rotated by 45° from their parallel position (Figure 1b). The SEM examination of the surface of the gladius blade (Figure 2a) shows a corrugated, fiber-like structure with channels running along the long axis of the gladius. Smaller fibers are observed on the surface of the larger, primary fibers. The cross section SEM image of the gladius (Figure 2b) shows a compact lamellar structure, with lamellae parallel to the gladius blade. Each lamella shows a thickness between 2 and 10 µm. These microscopic observations are in agreement with previous structural descriptions of the gladius reported by Hunt and El Sherief [21] and Yang *et al.* [18].

**Figure 3:**
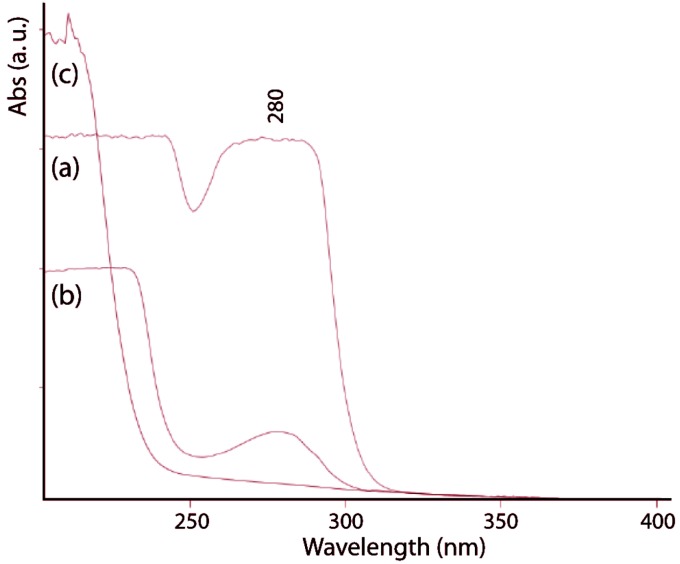
UV-vis spectra of untreated gladius sample (**a**) and samples treated in a boiling 1.0 M NaOH solution for 1 h (**b**) and 2 h (**c**). The band at 280 nm is diagnostic of the presence of proteins.

The protein content of the gladius was determined in 36% ± 2% (*w*/*w*), in agreement with values reported for other species [22,23]. The alkaline treatment causes deproteinization that was followed by UV-visible spectroscopy (Figure 3) recording the spectra between 200 and 400 nm. After 2 h of alkaline treatment, the absorption band at 280 nm, which is due to aromatic amino acids, completely disappears. The protein free gladius conserved shape and overall ultrastructure. Only a partial misalignment of the fibers, which makes the material no longer perfectly birefringent, was revealed by optical microscopy (Figure 1c). The alkaline treatment provokes a partial hydrolysis of the *N*-acetyl group. As a consequence, the degree of deacetylation (Table 1), evaluated by FTIR spectroscopy [24], increases with the time of treatment from 5% (2 h) up to 10% (24 h).

**Table 1:** Main features of samples of *Loligo vulgaris* gladius treated in reflux in a 1.0 M NaOH solution for different times: 2 h, 6 h and 24 h. For each measurement at least three samples from different gladii were analyzed.

	2 h	6 h	24 h
deacetylation (%)	5 ± 2	9 ± 2	10 ± 2
swelling (% *w*/*w*)	815 ± 20	760 ± 50	630 ± 35
contact angle (°)	81 ± 5	88 ± 8	93 ± 6
Young modulus (MPa)	273 ± 10	244 ± 15	227 ± 20
maximum deformation (%)	9 ± 3	10 ± 3	10 ± 3

**Figure 4:**
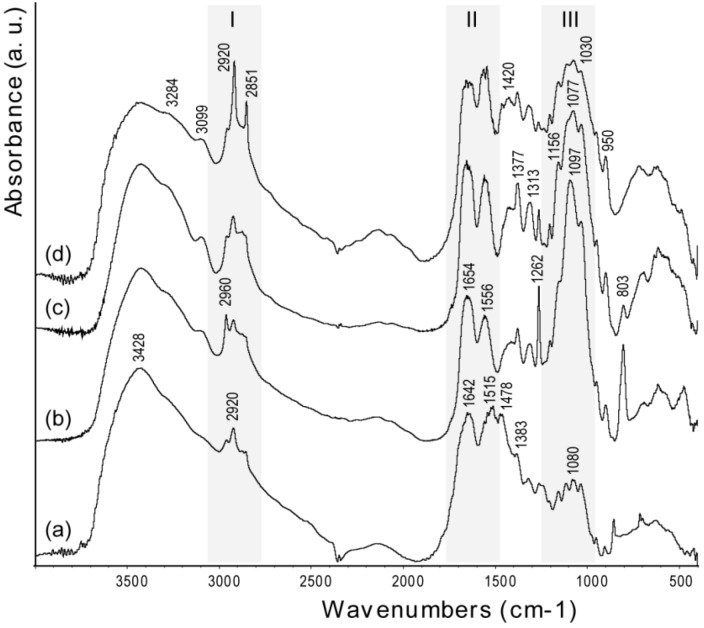
FTIR spectra of samples from *Loligo vulgaris* gladius. (**a**) Untreated sample; (**b**) Sample treated for 2 h in a boiling 1.0 M NaOH solution; (**c**) Sample treated for 6 h in a boiling 1.0 M NaOH solution; (**d**) Sample treated for 24 h in a boiling 1.0 M NaOH solution. The grey bars highlight the region where the characteristic absorption bands of alkyl groups (I), acetoamide groups (II) and saccharidic groups (III) were located. The wavenumber of each band is reported only once in the different spectra.

The bands in the FTIR spectra (Figure 4) of chitin samples were assigned according to literature [25]. The spectra exhibit a broad peak at 3428 cm^−1^, due to OH stretching, which becomes broader and shows shoulders at lower frequency, upon increase of the degree of deacetylation. This trend has been ascribed to structural disorder [26]. The amide I (1642 cm^−1^) and amide II (1515 cm^−1^) bands become weaker and shift to higher frequency after deproteinization (1654 and 1556 cm^−1^, respectively). This shift is consistent with the removal of the proteic component in α-coil conformation [18]. Increasing the degree of deacetylation, the polysaccharidic band, centered around 1100 cm^−1^, does not change its relative intensity with respect to the amide I band, but slightly changes its peak structure [27]. The relative intensity between the polysaccharidic band and the amide I band was determined by measuring the maximum of absorbance of each band after background correction. Interestingly, with an increase of deacetylation, *i.e.*, time of thermal treatment, the bands related to the methyl of the N-acetyl group (2851, 1377 and 950 cm^−1^) increase their intensity, as do the amide II and III bands at 1556 and 1313 cm^−1^, respectively. Analogously, there is an increase of the intensity of the bands at 2920 and 1420 cm^−1^ (-CH2 stretching) and at 1262 cm^−1^ (NH bending) [28]. The breaking of interchain interactions [13] allows water to percolate and partly hydrate the system. The strong water dipoles couple with local dipoles of the bonds, which, in turn, promotes an increase of the infrared intensities [29].

On the basis of the FTIR observations, the decrease of swelling and hydrophilicity associated to the deacetylation process (Table 1) appears more related to structural reorganization associated to the thermal treatment rather than to formation of free amino groups (deacetylation process) [30]. In this view, the small increase of deacetylation (from 5% to 10%) can be considered the trigger of the structural reorganization rather than a direct effect.

The structural reorganization of the β-chitin fibers was also evident at the microscale. SEM images in Figure 2 show that, as a consequence of the deacetylation process/thermal treatment, the samples become spongy. Pores separated by irregularly spaced lamellae are present and their size ranges from sub micrometers up to about 10 µm. The samples treated for 6 h and 24 h are qualitatively more porous than the sample treated for 2 h. This difference in porosity could not be explained only by the different degree of deacetylation, that changes from ~10% to ~5%. Thermal treatment must also break interchain chemical interactions. The SEM images also show that the surface features do not change for samples treated for different times. This observation, together with the presence of lamellae in the bulk of the samples, suggests that the interchain interactions are weakened by the alkaline digestion preferentially along the direction normal to the blade surface rather than in the parallel direction.

**Figure 5:**
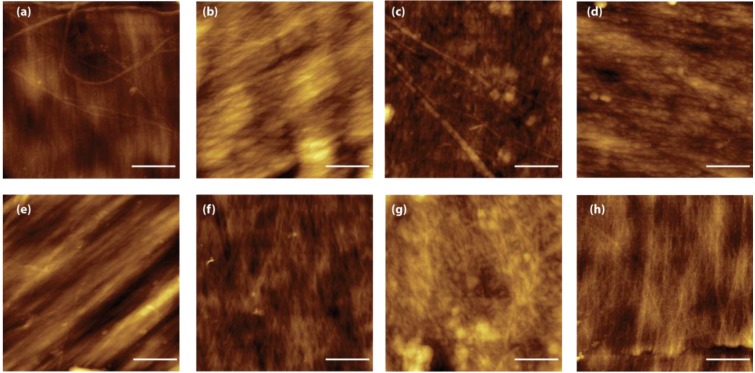
AFM images (2 µm × 2 µm) of samples from *Loligo vulgaris* gladius. (**a**,**e**) Untreated sample; (**b**,**f**) Sample treated for 2 h in a boiling 1.0 M NaOH solution; (**c**,**g**) Sample treated for 6 h in a boiling 1.0 M NaOH solution; (**g**,**h**) Sample treated for 24 h in a boiling 1.0 M NaOH solution. In all the images the scale bar is 500 nm.

The presence of a structural reorganization of the chitin fibers was also observed by AFM [31]. Figure 5 reports representative images of the blade surface after alkaline treatment. These images present the real topography and allow the measuring of surface parameters such as the roughness. Two kinds of topographies are observed in all the samples. In some regions, the fibers appear compact and well aligned (Figure 5a–c), in other regions the presence of micro- and nano-pores is observable. Although difficult to quantify, due to the heterogeneity of the samples, the AFM images show also a reduction of fiber thickness when the time of treatment is increased, as is expected in the case of a breaking of intermolecular linkages. This evidence was also reflected by the RMS roughness measured in the fiber areas over a 4 µm^2^ area; the values decrease from 7.8 ± 2.2 nm after 2 h of treatment, to 4.8 ± 0.4 nm and 4.4 ± 0.7 nm for the 6 h and 24 h of treatment, respectively. This trend can be explained by the presence of a hierarchical organization of the chitin fibers. Microfibers 0.2 µm sized have been reported as building blocks of larger chitin fibers [18].

**Figure 6:**
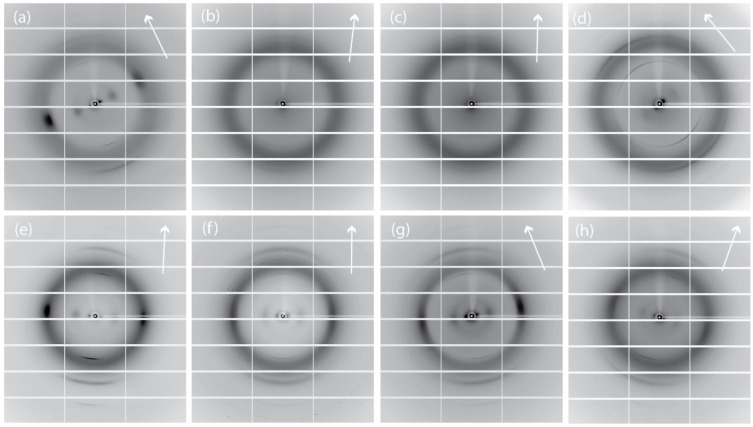
Synchrotron X-ray fiber diffraction diagrams of samples from *Loligo vulgaris* gladius. (**a**,**e**) Untreated sample; (**b**,**f**) Sample treated for 2 h in a boiling 1.0 M NaOH solution; (**c**,**g**) Sample treated for 6 h in a boiling 1.0 M NaOH solution; (**d**,**h**) Sample treated for 24 h in a boiling 1.0 M NaOH solution; (**a**–**d**) Wet samples; (**e**–**h**) Dry samples. The arrow indicates the meridian direction. The direction of the X-ray beam was perpendicular to the blade surface.

A view at the molecular scale of the structural changes induced by the alkaline treatment at different times was obtained by the analysis of the synchrotron X-ray diffraction data. The blade of the gladius was crystalline, as shown by its birefringent properties (Figure 1b). Figure 6a–d illustrate the X-ray fiber diffraction diagrams from wet samples that were never dried. The diagram from the untreated sample (Figure 6a) shows sharp reflections with some azimuthal distributions. All of the reflections could be indexed (Table 2) according to reported one-chain monoclinic unit cell (space group was P2_1_) with dimensions *a* = 4.8 Å, *b* = 11.1 Å, *c* (fiber repeat) = 10.44 Å, and γ (monoclinic angle) = 96.39°. For this hydrated β-chitin, one *N*-acetyl-d-glucosamine residue and two water molecules are contained in the asymmetric unit [32]. The deproteinization process decreases the intensity of the reflections in the X-ray diagrams and induces the appearance of a broad isotropic ring centered at about 4 Å, associable to X-ray scattering from amorphous material. The sample treated for 2 h (Figure 6b) shows a diffraction diagram where only weak meridian (002) and (013) reflections are observed; the sample treated for 6 h (Figure 6c) shows an additional very weak equatorial (010) reflection. The wet sample treated for 24 h show a diffraction diagram containing a medium intensity meridian (002) reflection and weak meridian (013) and (004), and equatorial (010), (100), (1ī1) and (110), reflections. The meridian (002) reflection shows an increasing azimuthal distribution with the increase of the treatment time. These variations indicate an increasing misalignment of the chitin chains along the fiber direction. The equatorial (010) reflection, when present, broadens and increases its periodic distance (from 11.1 to 12.7 Å). All the other reflections do not change their periodic distances (Table 2). This finding indicates that, as a consequence of the treatment, the *b* parameter of the β-chitin unit cell expands laterally, whereas *a* and *c* remain constant. The trend can be associated to the incorporation of water molecules inside the crystalline lattice. A similar structural effect was reported for the incorporation of several swelling agent in the β-chitin lattice [33]. As mentioned above, no inter-sheet hydrogen bond is present in the crystal structure of β-chitin, whereas the sheets themselves are tightly bound by a number of intra-sheet hydrogen bonds.

**Figure 7:**
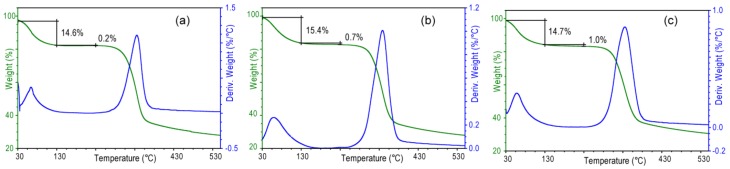
Thermogravimetric profiles of air dried samples from *Loligo vulgaris* gladius after alkaline digestion in a 1.0 M NaOH solution for 2 h (**a**), 6 h (**b**) or 24 h (**c**). Water is lost in two distinct steps. From about 50 to 130 °C, the first step is assigned to loosely bound water. In the second step from 130 to 250 °C, structural water is lost.

The wet samples were air dried. In Figure 6e–h, the corresponding X-ray fiber diffraction diagrams are illustrated. The diagrams show a different distribution of reflection intensities than in the case of the wet samples. The untreated samples show more reflections. On the meridian of the diagram, the (001) reflection is very weak, (002) is strong, (013) is medium, and (004) is weak. The presence of the (001) reflection, forbidden by symmetry, indicates a certain degree of structural disorder, also mirrored by the azimuthal distribution of the meridian reflections. On the equatorial direction, the (010), (100), (1ī1) and (110) reflections show a distribution of intensities similar to those observed in the wet sample. The periodic distances do not change with respect to those observed in the wet sample. This finding indicates that the β-chitin fibers of the untreated samples, covered by a proteic wrap, do not change their hydration state upon air drying. The dry treated samples show a general increase of the intensity of the reflections and a weakening of the isotropic ring with respect to the wet ones. Only the periodicities associated to the equatorial (010) reflection change and are shorter (Table 2) than those in the corresponding wet samples. The analysis of these values indicates that in the samples subject to a longer treatment a higher amount of water remains entrapped between the sheets of chitin after drying. This feature was confirmed by the thermogravimetrical analysis (Figure 7) that shows that the content of structural water, evaluated by the percentage of weight lost between 130 and 230 °C, increases from 0.2% (sample 2 h) to 1.0% (sample 24 h). It is worth to note that in all the samples the meridian reflections are always observed, indicating that the crystalline order along the fiber axis (*c* axis) is always present. Thus, the alkaline treatment affects mainly the lateral packing, or association, of the chitin fibers.

The results of mechanical tests on the wet samples are in line with the above observations (Table 1). When the samples are uniaxially deformed along the fiber axis, they show a reduction of the Young modulus, from 273 ± 10 to 227 ± 20 MPa, going from the sample treated 2 h to that treated 24 h, while the maximum deformation remains unchanged (about 10%). Accordingly, the decrease of the Young modulus is associated to the reduction of the side interactions between the chitin fibers, which does not affect the maximum deformation [34].

**Table 2:** List of reflection from 2D X-ray diffraction diagrams collected from samples of *Loligo vulgaris* gladius untreated (**wt**) and treated in reflux with a 1.0 M NaOH solution for different times: 2 h, 6 h and 24 h. The reflections of the untreated samples are indexed according to a monoclinic unit cell (*a* = 4.85 Å, *b* = 9.26 Å, *c* = 11.1 Å (fiber axis), and γ = 97.5°; space group P2_1_). The periodicity associated to each reflection is indicated in Å and the relative intensity is indicated in parenthesis.

	wt		2 h		6 h		24 h	
	*wet*	*Dry*	*wet*	*dry*	*wet*	*dry*	*wet*	*dry*
(010)	11.1 (m)	11.1 (m)	-	12.0 (m)	12.4 (vw)	12.0 (w)	12.7 (w)	12.3 (w)
(100) (1ī1)	4.6 (s)	4.6 (s)	-	4.6 (m)	-	4.6 (s)	4.6 (vw)	4.6 (w)
(110)	4.2 (m)	4.2 (w)	-	4.2 (ww)	-	4.2 (ww)	4.2 (vw)	4.2 (vw)
(001)	-	10.1 (vw)	-	10.1 (vw)	-	10.1 (vw)	-	10.1 (w)
(002)	5.1 (m)	5.1 (s)	5.1 (w)	5.1 (m)	5.1 (w)	5.1 (m)	5.1 (m)	5.1 (m)
(013)	3.3 (w)	3.3 (m)	3.3 (vw)	3.3 (w)	3.3 (w)	3.3 (m)	3.3 (s)	3.3 (m)
(004)	2.56 (vw)	2.56 (w)	-	2.56 (vw)	-	2.56 (w)	2.56 (vw)	2.56 (vw)
a. r.	4.0 (vw)	4.2 (vw)	4.0 (s)	4.2 (w)	3.7 (m)	4.2 (w)	3.7 (m)	4.4 (vw)

The relative intensity of the reflections is scaled as strong (s), medium (m), weak (w) and very weak (wv). a. r. indicates amorphous ring. - indicates the absence of a detectable reflection.

## 3. Experimental Section

Fresh *Loligo vulgaris* gladii were obtained from the local fish market in Bologna, Italy. The gladii were about 20 cm in length and about 5 cm in width. The gladii were washed thoroughly with tap water to remove soluble organics and adherent proteins. The fresh gladius started to dry quickly in air, so that initial experiments did not give conclusive results since the structure kept changing in time and cracks appeared on the blade surface. For this reason the gladii were never dried, unless required for specific experiments, and stored in distilled water containing sodium azide. The gladii were then stable for several weeks.

The experimental characterization and the chemical treatment were carried out on pieces 6 cm long and 1 cm wide from the central part of the blade. Dried samples were prepared by freeze drying.

The chitin from the gladii was purified from proteins and partially deacetylated as follows. They were first refluxed in 1.0 M NaOH aqueous solution for 2 h, 6 h or 24 h followed by several rinsing in distilled water until the pH was close to neutrality.

The average content of chitin (*n* = 6) in the gladius was determined by measuring the weight of dry pieces of gladii before and after the 1 hour treatment with a boiling 1.0 M NaOH solution.

The degree of deacetylation (DDA) was evaluated by FTIR spectroscopy because of its simplicity, but this methodology requires a calibration [27], such as the elemental analysis used here. The DDA of six gladii samples treated at different times was determined by elemental analysis with at least three measurements for each sample. The FTIR spectrum was recorded and the intensity ratio between the band at 1313 cm^−1^ (Amide III), and that at 1420 cm^−1^ (-CH_2_ bending), was plotted as a function of the DDA determined by elemental analysis. A calibration curve was obtained by linear regression analysis of the data and used to determine the DDA by FTIR, the error was calculated applying the theory of error propagation.

The swelling measurements were carried out on square-shaped samples (1 cm^2^) immersed in distilled water for different periods of time. The wet samples were wiped with filter paper to remove excess liquid and weighted. The amount of adsorbed water was calculated as the percentage of increase of weight of the wet sample with respect to the weight of the dry sample. At least three specimens were measured for each sample type and results were provided as the average value ± error.

Fourier transform infrared (FTIR) spectroscopy analyses were collected using a FTIR Nicolet 380 instrument (Thermo Electron Co., Waltham, MA, USA) from 4000 to 400 cm^−1^ at a resolution of 4 cm^−1^. Disks were made by applying a pressure of 48.6 psi to a mixture consisting of 1 mg of sample and 100 mg of KBr by means of a hydraulic press. Prior to the measurements the samples were dried in a dessicator containing P_2_O_5_.

The static contact angle was measured using a Contact Angle meter DGD-DX model (DIGIDROP, Bourg de Peage, France).

AFM measurements were carried out using a Multimode VIII equipped with a Nanoscope V (Bruker Nano Surface, Palaiseau, France). The microscope was operated in PeakForce Tapping mode and all the images were collected at the same scan rate (1 Hz). The cantilever used were SCANASYST-AIR-HR (Bruker Nano Surface, Palaiseau, France) with a nominal spring constant of 0.4 N/m. The real spring constant was anyway measured by thermal tuning for each cantilever. The gladius portions were cutted and then fixed onto a magnetic sample holder using a suitable double side gluing disks. All measurements were performed in air or under a nitrogen flow to control the humidity. Electron microscopy investigations were performed using a Phenom™ scanning electron microscope.

X-ray diffraction diagrams were collected at XRD1 beamline, Elettra, Trieste, Italy. Each frame was collected at the peak wavelength (0.9999 Å) using an exposure of 60 s. The wet samples were collected in cryogenic condition (30% glycerol solution) at the temperature of 100 K. The X-ray diffraction diagrams were analyzed using the software Fit2D [35].

Thermogrametric investigations were carried out on dried samples using Instruments SDT 2960 at a heating rate of 10 °C/min in a nitrogen atmosphere over a temperature range from 30 to 600 °C. Sample weights were 3–5 mg, and the nitrogen flow rate was 100 mL/min.

Mechanical characterization was carried out on strip shaped (6–60 mm, thickness around 0.2 mm) wet samples. Stress-strain curves were recorded on wet samples using an INSTRON Testing Machine 4465, and the Series IX software package. Crosshead speed was set at 5 mm/min. The Young’s modulus and the strain at break of the strips were measured in a static mode. At least three specimens were measured for each sample type and results were provided as the average value ± error.

## 4. Conclusions

In conclusion this work showed that porosity, wettability and swelling of β-chitin materials from the *Loligo vulgaris* gladius can be controlled by alkaline treatments, using a *top up* approach. Moreover, it was observed that the alkaline treatment changes the chemical environment of the chitin *N*-acetyl groups, favoring the intercalation of water molecules between the chitin sheets and changing the crystalline packing of β-chitin. The potential applications of these β-chitin materials range from nanotechnology to regenerative medicine. It is worth mentioning that the use of gladii, which are waste products of the fishing industry, has also important environmental implications.
